# Comprehensive insights into transcriptional adaptation of intracellular mycobacteria by microbe-enriched dual RNA sequencing

**DOI:** 10.1186/s12864-014-1197-2

**Published:** 2015-02-05

**Authors:** Rienk A Rienksma, Maria Suarez-Diez, Hans-Joachim Mollenkopf, Gregory M Dolganov, Anca Dorhoi, Gary K Schoolnik, Vitor AP Martins dos Santos, Stefan HE Kaufmann, Peter J Schaap, Martin Gengenbacher

**Affiliations:** Laboratory of Systems and Synthetic Biology, Wageningen University and Research Centre, Dreijenplein 10, 6703 HB Wageningen, the Netherlands; Core Facility Microarray/Genomics, Max Planck Institute for Infection Biology, Charitéplatz 1, 10117 Berlin, Germany; Department of Microbiology and Immunology, Stanford University School of Medicine, 300 Pasteur Drive, Stanford, CA 94305-5124 USA; Department of Immunology, Max Planck Institute for Infection Biology, Charitéplatz 1, 10117 Berlin, Germany; LifeGlimmer GmbH, Markelstrasse 38, 12163 Berlin, Germany; Present address: Department of Microbiology, Yong Loo Lin School of Medicine, National University of Singapore, 5 Science Drive 2, Singapore, 117545 Singapore

**Keywords:** *Mycobacterium bovis* BCG, THP-1 cells, Infection, Host-microbe interaction, Transcriptome, Dual RNA sequencing, Microbe enrichment

## Abstract

**Background:**

The human pathogen *Mycobacterium tuberculosis* has the capacity to escape eradication by professional phagocytes. During infection, *M. tuberculosis* resists the harsh environment of phagosomes and actively manipulates macrophages and dendritic cells to ensure prolonged intracellular survival. In contrast to other intracellular pathogens, it has remained difficult to capture the transcriptome of mycobacteria during infection due to an unfavorable host-to-pathogen ratio.

**Results:**

We infected the human macrophage-like cell line THP-1 with the attenuated *M. tuberculosis* surrogate *M. bovis* Bacillus Calmette–Guérin (*M. bovis* BCG). Mycobacterial RNA was up to 1000-fold underrepresented in total RNA preparations of infected host cells. We employed microbial enrichment combined with specific ribosomal RNA depletion to simultaneously analyze the transcriptional responses of host and pathogen during infection by dual RNA sequencing. Our results confirm that mycobacterial pathways for cholesterol degradation and iron acquisition are upregulated during infection. In addition, genes involved in the methylcitrate cycle, aspartate metabolism and recycling of mycolic acids were induced. In response to *M. bovis* BCG infection, host cells upregulated *de novo* cholesterol biosynthesis presumably to compensate for the loss of this metabolite by bacterial catabolism.

**Conclusions:**

Dual RNA sequencing allows simultaneous capture of the global transcriptome of host and pathogen, during infection. However, mycobacteria remained problematic due to their relatively low number per host cell resulting in an unfavorable bacterium-to-host RNA ratio. Here, we use a strategy that combines enrichment for bacterial transcripts and dual RNA sequencing to provide the most comprehensive transcriptome of intracellular mycobacteria to date. The knowledge acquired into the pathogen and host pathways regulated during infection may contribute to a solid basis for the deployment of novel intervention strategies to tackle infection.

**Electronic supplementary material:**

The online version of this article (doi:10.1186/s12864-014-1197-2) contains supplementary material, which is available to authorized users.

## Background

Tuberculosis (TB) is an infectious disease caused by the airborne pathogen *Mycobacterium tuberculosis* and accounts for 1.3 million fatalities annually [1]. Unlike non-pathogenic microbes that are eliminated inside the maturing phagosome of immune cells such as macrophages, *M. tuberculosis* brings phagosome maturation to a halt and manages to cope with various host threats including acidification, reactive radicals and nutrient limitation [2]. Studying the transcriptome of intracellular pathogens, in particular *M. tuberculosis*, during infection remained difficult due to a low bacteria-to-host RNA ratio. For different pathogens the number of organisms per host cell spans several orders of magnitudes ranging from 1 to 10 for *M. tuberculosis* and up to 1000 for *Chlamydia* [3,4].

The first insights into the intracellular life of *M. tuberculosis* provided by comparative microarray analysis, revealed a switch from aerobic to anaerobic respiration, induction of the dormancy regulon *dosR* and iron scavenging as well as upregulation of β-oxidation of fatty acids upon infection [5]. Similar technologies and quantitative real-time PCR were applied to broaden our understanding of specific aspects of intracellular *M. tuberculosis* [6-9]. Microarray probes have the disadvantage of unspecific cross-hybridization between pathogen and host [4], and most often such probes are not optimized for minimal cross-reactivity with other species. Cappelli and colleagues [8] estimated that non-specific signals account for up to 12.5% of all signals. Additionally, transcription of non-coding regions and missed or miss-annotated genes often remain disregarded due to a limited array design. Quantitative real-time PCR has mostly been applied to small subsets of genes, since detection of each transcript requires a pair of specific oligonucleotides [6-9].

Dual RNA sequencing (dual RNA-seq) is a relatively novel technique to study gene expression profiles. This technique allows unbiased and simultaneous sequencing of transcriptomes of multiple organisms and therefore is a superb technology to study intracellular pathogens during infection of host cells. The sequencing reads can subsequently be matched *in-silico* to the respective organism. Without prior knowledge of sample content, its composition can be deduced from dual RNA-seq datasets without targeting specific species [10]. Most importantly, dual RNA-seq captures the transcriptome in its entirety thereby overcoming the limitations of microarrays discussed above. First application of this technology to study *M. avium* subsp. *paratuberculosis* during macrophage infection has shed new light on mycobacterial iron acquisition [11].

The attenuated TB vaccine strain *M. bovis* Bacillus Calmette–Guérin (*M. bovis* BCG) has been widely used in research as surrogate for pathogenic *M. tuberculosis* due to a high degree of genome identity [12-14]. In this study, we investigated the transcriptional adaptation of *M. bovis* BCG 24 hours after infection of the human macrophage-like cell line THP-1 by dual RNA-seq. The underrepresentation of bacterial RNA in preparations of total RNA from infected host cells requires high sequencing depth to gain statistical significance and adequate pathogen coverage, leading to increased costs. Mangan and colleagues developed a method entailing differential lysis with guanidine thiocyanate to enrich for mycobacteria from infected macrophages, thus avoiding massive underrepresentation of bacterial RNA as compared to total RNA preparations of infected cells [15]. This method has been used for *in vivo* transcriptome studies using microarrays [6,16]. Here we present a strategy that combines bacterial enrichment for bacterial transcripts and dual RNA-seq, which we evaluate against non-enriched samples.

## Results

**Figure 1:**
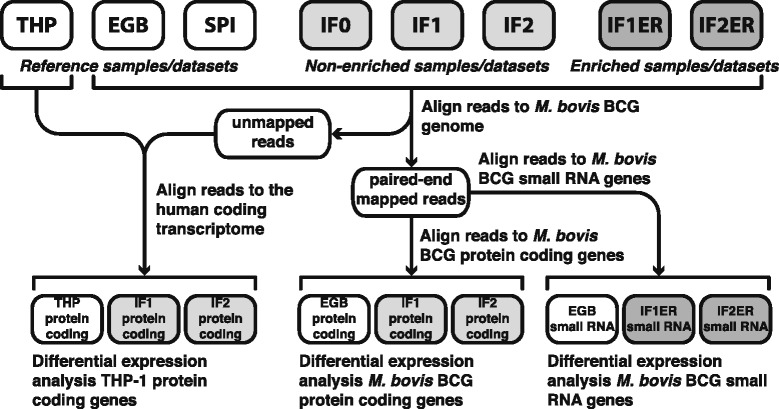
**Schematic overview of RNA sequencing data analysis.** A total of eight datasets were processed by aligning the 50-bp paired-end sequencing reads to the human transcriptome, the *M. bovis* BCG genome, and/or the *M. bovis* BCG gene and small RNA sequences. Six of these datasets were used for differential gene and/or small RNA expression analysis. (THP: Reference dataset for the THP-1 transcriptome, EGB: Reference dataset for the (exponentially growing) *M. bovis* BCG transcriptome, SPI: Spike-in dataset, IF0/1/2: Datasets of *M. bovis* BCG bacilli infecting THP-1 cells, IF1/2ER: Datasets of *M. bovis* BCG cells infecting THP-1 cells enriched for *M. bovis* BCG bacilli).

Twenty-four hours post-infection, THP-1 cells were harvested and total RNA was isolated. Additionally, two out of three infected THP-1 samples were enriched for *M. bovis* BCG bacilli, using the procedure described in Methods. The analysis of the 50-bp RNA-derived paired-end sequencing data is illustrated in Figure 1. Two out of the three datasets derived from the non-enriched infections (IF1/2) were compared to a reference sample with uninfected THP-1 cells (THP) and differentially expressed THP-1 genes were identified. For differential *M. bovis* BCG gene and small RNA expression analysis, the datasets derived from enriched infections (IF1/2ER) were compared to a reference culture of exponentially growing *M. bovis* BCG (EGB). A spike-in sample (SPI) was used to estimate the percentage of infected cells and to correlate the reads of spiked-in *M. bovis* BCG with the *M. bovis* BCG culture and the non-enriched THP-1 infections with *M. bovis* BCG. An overview of the primary sequencing data is depicted in Table 1.

**Table 1:** **Reads (millions and percentages) mapped on the human transcriptome and the** ***M. bovis*** **BCG genome**

		**Human transcriptome**		***M. bovis*** **BCG genome**		
**Dataset**	**Description**	**M of reads**	**%**	**M of reads**	**%**	**Total**
THP	Uninfected THP-1 cells	30.9	100	–	–	30.9
EGB	*M. bovis* BCG bacilli	–	–	168	100	168
SPI	Mixed THP-1 and *M. bovis* BCG RNA	31.8	91.0	3.16	9.0	35.0
IF0	Infected THP-1 cells replicate 0	21.5	98.0	0.45	2.0	21.9
IF1	Infected THP-1 cells replicate 1	38.2	96.0	1.57	4.0	39.7
IF2	Infected THP-1 cells replicate 2	28.0	96.3	1.07	3.7	29.0
IF1ER	Infected THP-1 cells replicate 1 enriched for *M. bovis* BCG bacilli	18.0	74.7	6.09	25.3	24.0
IF2ER	Infected THP-1 cells replicate 2 enriched for *M. bovis* BCG bacilli	26.0	88.6	3.35	11.4	29.4

### Pathogen specific enrichment strategy is effective

It has been estimated that a minimum of 2–5 million reads from a ribosomal RNA-depleted library is required to adequately cover the gene expression profile of a pathogen in a dual RNA-seq experiment [17-19]. Datasets IF0, IF1 and IF2, derived from non-enriched infections contained 0.4, 1.6 and 1.1 million 50-bp reads that aligned to the *M. bovis* BCG genome, which was too low for significant coverage of the gene expression profile. Subsequently, an enrichment strategy for *M. bovis* BCG was applied to overcome this obstacle, thereby increasing the coverage of intracellular *M. bovis* BCG transcripts. These enriched datasets (IF1ER and IF2ER) contained 6.1 and 3.3 million 50-bp reads that aligned to the *M. bovis* BCG genome (Table 1).

**Figure 2:**
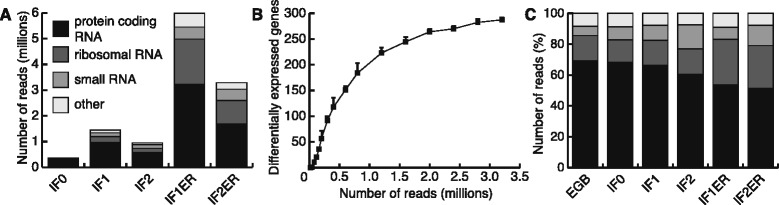
**Classification of 50-bp sequencing reads and effect of increasing sequencing depth. (A)** The total number of 50-bp sequencing reads, matching the paired-end analysis criterion that both reads could be aligned to the *M. bovis* BCG genome, were assigned to four different categories (protein-coding RNA, ribosomal RNA, small RNA, and other). The total of the reads for each sample represents the number of reads aligning to the *M. bovis* BCG genome. **(B)** Simulation of the relation between the number of differentially expressed *M. bovis* BCG genes and sequencing depth. Random subsets of reads were selected from EGB, IF1ER and IF2ER and the mean number (n = 5) of reliably identified differentially expressed genes (FDR < 0.05) and the standard deviation (error bars) are given for various sequencing depths. Note that the ratio of a random set to the total set approaches 1 as the size of the random set increases. Therefore, the random samples become more similar to each other and the standard deviation decreases. For reasons of completeness, we have included a standard deviation for every point. **(C)** Classification of the relative number of 50-bp paired-end sequencing reads aligning to the *M. bovis* BCG genome. The legend is the same as in **(A)**.

The absolute number of *M. bovis* BCG reads of all infected sample preparations was subsequently classified in four different categories: protein-coding RNA, ribosomal RNA, small RNA, and other (Figure 2A). We simulated the relationship between the number of identified differentially expressed protein-coding *M. bovis* BCG genes and sequencing depth (Figure 2B). For very low numbers of sequencing reads, the number of identified genes increases in a linear way with the library size. With increasing library size the number of correct identifications tends to stabilize (Figure 2B). The relative abundance of the four different categories was fairly similar in both, enriched and non-enriched samples, demonstrating that impact of enrichment per se on *M. bovis* BCG derived sequencing reads is negligible (Figure 2C). The normalized counts of the protein coding *M. bovis* BCG transcripts in the enriched datasets (IF1ER and IF2ER) and the non-enriched datasets (IF1 and IF2) revealed a linear relationship, with Pearson’s correlation coefficients of 0.91 and 0.92, respectively (Additional file 1). We conclude that pathogen enrichment does not introduce any bias to protein-coding gene expression of *M. bovis* BCG. However, the correlation for normalized counts per gene of THP-1 reads between the same datasets is much lower, 0.57 and 0.70, respectively (Additional file 1). Therefore, the non-enriched datasets (IF1 and IF2) were used for differential gene expression analysis of THP-1 genes. This enrichment procedure thus enabled us to study the intracellular gene expression of *M. bovis* BCG during infection.

### *M. bovis* BCG response to infection

Twenty-four hours post-infection a clear response of the phagocytosed *M. bovis* BCG bacilli can be observed on the transcriptome level. A total of 367 *M. bovis* BCG genes were differentially expressed (FDR < 0.05), of which 216 were induced and 151 were repressed. A list of all differentially expressed genes of both *M. bovis* BCG and THP-1 cells is provided in Additional file 2.

### *M. bovis* BCG cholesterol catabolism genes are induced during infection

Cholesterol is a complex lipid that consists of three cyclohexane rings (A, B and C), a cyclopentane ring (D), and an 8-carbon side chain. An incomplete degradation pathway of cholesterol was recently proposed for *M. tuberculosis* [20]. This pathway was extended with the side chain degradation of rings C and D (Additional file 3) and several genes involved in the pathway were added based on additional literature [21-28]. This extended cholesterol degradation pathway has been previously described in a genome-scale metabolic model of *M. tuberculosis* [29].

**Figure 3:**
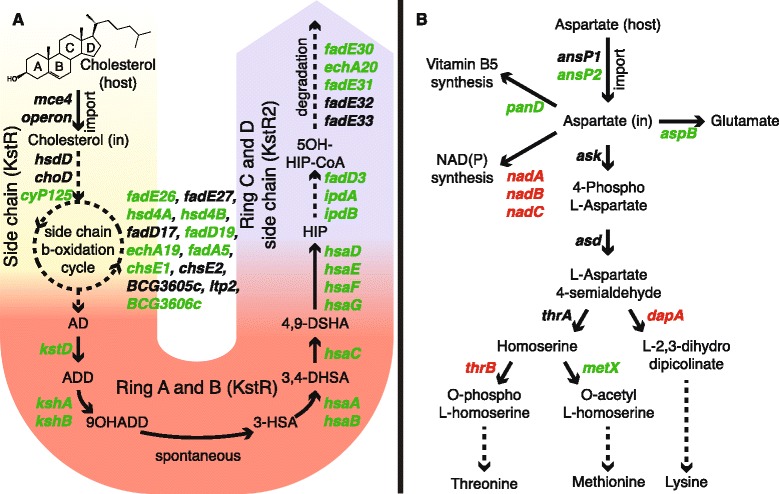
**Metabolic processes during infection.** Genes in green are induced upon infection (FDR < 0.05), genes in red are repressed (FDR < 0.05) and genes in black show no differential expression. **(A)** Cholesterol degradation is divided in three parts: The degradation of the side chain (yellow part), degradation of rings A and B (red part) and the degradation of the side chain of rings C and D (blue part). Dashed arrows represent multiple reactions. The degradation of the rings C and D side chain is based on homologous genes from *Rhodococcus equi*. AD: 4-androstenedione, ADD: 1,4-androstenedione, 9OHADD: 9-hydroxy-1,4, androstene-3-17-dione, 3-HSA: 3-hydroxy-9,10-seconandrost-1,3,5(10)-triene-9,17-dione 3,4-DHSA: 3,4-dihydroxy-9,10-seconandrost-1,3,5(10)-triene-9,17-dione 4,9 DSHA: 4,5-9,10-diseco-3-hydroxy-5,9,17-trioxoandrosta-1(10),2-diene-4-oic acid, HIP: 9,17-dioxo-1,2,3,4,10,19-hexanorandrostan-5-oic acid, 5OH-HIP: 5-hydroxy-methylhexahydro-1-indanone propionate. **(B)** Aspartate could be imported via AnsP2 and used for the synthesis of vitamin B5, glutamate and methionine. *thrB*, *dapA* and *nadABC* are downregulated, indicating that aspartate is to a lesser extent used to synthesize threonine, lysine and NAD(P).

We observed a strong increase in expression of almost all genes assigned to cholesterol degradation (Figure 3A and Additional file 3). Initially, cholesterol is taken up by the transport system encoded by the *mce4* gene cluster [30]. The 3β-hydroxyl group is oxidized and isomerized to cholest-4-en-3-one either by the membrane-bound oxidase ChoD or by the dehydrogenase HsdD [21,31]. No apparent induction of the *mce4* operon, the hydroxysteroid dehydrogenase (HsdD) and cholesterol oxidase (ChoD) coding genes was observed in our datasets. However, the number of transcript reads assigned to the *mce4* operon and to *choD* and *hsdD* indicate that they were expressed in both the infectious and the non-infectious state (Data set S1).

Although the degradation of rings A and B is well established, the side chain degradation of rings C and D (Figure 3A and Additional file 3) is less understood in mycobacteria and therefore was reconstructed based on orthology with *Rhodococcus equi* genes [22,26].

KstR and KstR2 (BCG3639, BCG3621c; Rv3574, Rv3557c) have been previously identified as regulators of cholesterol utilization in mycobacteria [32]. The KstR2 regulon comprises *kstR2* itself and all genes linked to the degradation of the side chain of rings C and D, whereas genes regulated by KstR participate in the degradation of rings A and B and the initial degradation of the cholesterol side-chain (Figure 3A and Additional file 3). In our datasets the expression of *kstR2* was strongly induced upon infection, whereas *kstR* remained unchanged (Data set S1). To verify these findings and the expression of other genes we selected a subset of 14 genes, of which 3 encode small RNAs, and designed primers (Additional file 4) to use for qRT-PCR. Among the selected genes, 5 genes are involved in cholesterol catabolism and 2 genes encode enzymes of the methylcitrate cycle (Additional file 5). The qRT-PCR results confirmed the integrity of our RNA-seq data.

**Figure 4:**
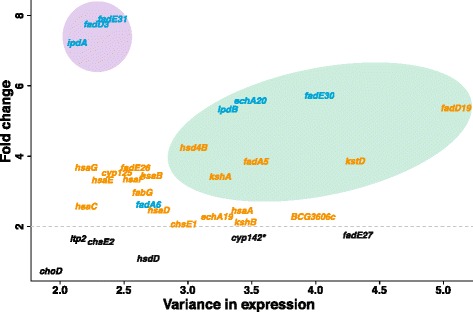
**Variance in expression levels (compendium) compared to the fold changes upon infection.** Genes in blue are KstR2-regulated whereas orange genes denote KstR-regulated genes. Genes in black show no significant change (FDR > 0.05). Green/violet ellipses denote areas of high/low variability in the expression compendium. *In *M. tuberculosis* H37Rv, *cyp142* has the same function as *cyp125*. In *M. bovis* BCG, *cyp142* encodes an inactive product.

**Figure 5:**
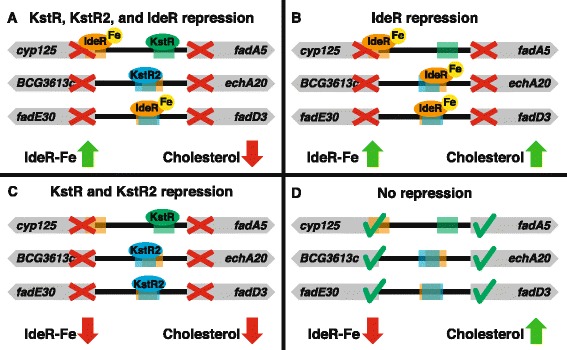
**Regulation of the cholesterol degradation pathway by IdeR, KstR and KstR2.** Sequences similar to the IdeR binding boxes appear in the upstream regions of genes in the cholesterol degradation pathway in close proximity to (and sometimes overlapping with) the KstR and KstR2 binding regions. **(A, B and C)** Under either normal iron availability or lack of cholesterol either IdeR or KstR/KstR2 represses the expression of genes in this pathway. **(D)** Only under low iron availability (relieving IdeR repression) and in presence of cholesterol (relieving KstR and KstR2 repression), can the genes in the cholesterol degradation pathway be expressed.

We analyzed the behavior of the genes in the cholesterol degradation pathway in a compendium of expression data collected for *M. tuberculosis*. Although no condition associated with cholesterol utilization has been included in the compendium, many conditions in our compendium lead to differential expression of genes regulated by KstR and KstR2 (Figure 4). Yet, a reduced set of KstR2-regulated genes (*fadD3*, *fadE31* and *ipdA*) exists, which seems to be specifically induced upon infection and most likely specifically reacts to only this kind of perturbation. The specific induction renders *fadD3*, *fadE31* and *ipdA* of potential interest for therapeutic intervention. Bioinformatics analysis using the consensus IdeR binding motif [33] and the KstR2 binding motif [32] revealed that these regions overlap (Figure 5).

Griffin and co-workers [34] found that although propionyl-CoA can be derived from other host metabolites, the requirement for the methylcitrate cycle is largely attributable to the degradation of host cholesterol. The induction of the methylcitrate cycle and the slight repression of *icd1* (BCG3409c; Rv3339c), encoding an isocitrate dehydrogenase, suggests that the oxidative part of the citric acid cycle is bypassed in favor of this pathway (Additional file 6). This emphasizes that cholesterol is the main carbon source for intra-phagosomal *M. bovis* BCG.

### Expression profile suggests *M. bovis* BCG recycles mycolic acids

**Figure 6:**
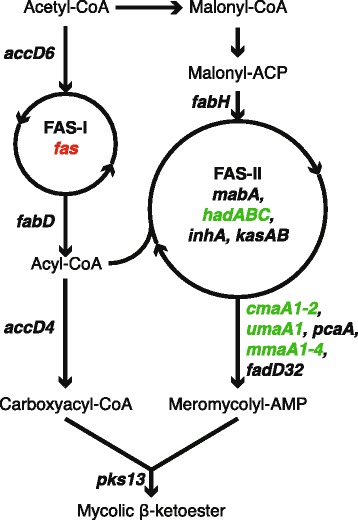
***M. bovis*** **BCG gene expression pattern of mycolic acid synthesis.** Genes involved in fatty acid synthase II (FAS-II) and downstream of FAS-II are induced (green), while fatty acid synthase I (FAS-I) is repressed (red).

Mycobacterial fatty acids are precursors for mycolic acids and are synthesized by at least two fatty acid synthases: FAS-I and FAS-II [35]. FAS-I consists of a single multifunctional enzyme, encoded by *fas* (BCG2545c; Rv2524c), and elongates fatty acids at the beginning of the mycolic acid synthesis pathway, while FAS-II consists of multiple enzymes and elongates fatty acids created by FAS-I. The mycobacterial genes *umaA1*, *cmaA2, hadA*, and *mmaA3* (BCG0509, BCG0546c, BCG0684, BCG0692c; Rv0469, Rv0503c, Rv0635, Rv0643) encode enzymes that further process FAS-II products (Figure 6). Previous reports suggested that FadE23 and FadE24 (BCG3163, BCG3162; Rv3140, Rv3139) might be involved in recycling of mycolic acids [24]. Taken together, the expression patterns observed in our study (Figure 6) indicate that new acids are rather generated by remodeling existing mycolic acids and host fatty acids than synthesized *de-novo*.

### Expression pattern of intracellular *M. bovis* BCG suggests utilization of host aspartate

Gouzy and colleagues showed that nitrogen incorporation from exogenous aspartate is required for host colonization by *M. tuberculosis* [36]. We observed changes in the gene expression pattern upon infection, regarding aspartate metabolism (Figure 3B). Intriguingly, the gene encoding the unique aspartate importer AnsP1 (BCG2144; Rv2127) showed no significant change in expression, while its homolog *ansP2* (BCG0385c; Rv0346c) showed a two-fold induction (Data set S1). Gouzy and colleagues found that an *M. tuberculosis ansP2*-knock-out (KO) mutant was able to grow on aspartate as sole nitrogen source [36]. Moreover, an *ansP1* mutant showed no growth defect in either resting or activated macrophages. The lack of induction of the sole asparaginase gene *ansA* (BCG1590c; Rv1538c), that can catalyze the conversion of asparagine to aspartate, suggests that, in addition to its reported asparagine transport capacity [37], mycobacterial AnsP2 imports aspartate from the phagosome during infection. Some of the genes that encode aspartate-utilizing enzymes are induced, such as *panD* and *aspB* (BCG3665c, BCG3629; Rv3601c, Rv3565). In particular, AspB was predicted to transfer nitrogen from aspartate to glutamate, which serves as a central nitrogen carrier for alternative metabolic pathways [38], suggesting that *M. bovis* BCG utilizes host aspartate as nitrogen source during infection.

The repression of *de novo* NAD(P) synthesis genes *nadA*, *nadB* and *nadC* (BCG1632, BCG1633, BCG1634; Rv1594, Rv1595, Rv1596) and the absence of significant changes in expression of *pncA*, *pncB*, *nadD* and *nadE* (BCG2062c, BCG1392c, BCG2437c, BCG2457c; Rv2043c, Rv1330c, Rv2421c, Rv2438c) involved in NAD(P) synthesis and salvage [39] (Data set S1), indicates that bacterial NAD(P) may become limited during infection. The transcripts of enzymes catalyzing branching reactions towards threonine, methionine and lysine showed an unexpected pattern (Figure 3B): both *dapA* (BCG2769c; Rv2753c) and *thrB* (BCG1356; Rv1296), involved in initiation of threonine and lysine biosynthesis respectively were repressed, while *metX* (BCG3411; Rv3341), encoding an enzyme that initiates methionine biosynthesis, was induced. We conclude that host aspartate utilized by *M. bovis* BCG might largely be converted into methionine rather than threonine and lysine.

The induction of *sodA* (BCG3909; Rv3846) (Data set S1), encoding superoxide dismutase that destroys harmful radicals, confirms that *M. bovis* BCG counteracts reactive oxygen intermediates (ROI) produced by the host cell [2,40]. Interestingly, aspartate has the capacity to quench ROI by intramolecular oxidation of the sulphur atom [41]. Although experimental prove has yet to be provided, it is attractive to speculate that mycobacteria produce methionine during infection to support counteraction to ROI.

### *M. bovis* BCG iron scavenging; siderophore synthesis, secretion and import

Mycobactins comprise an essential class of mycobacterial siderophore molecules to access iron of the host. These molecules are synthesized by an array of mycobactin enzymes, consisting of several proteins organized in a megasynthase [42]. The mycobactin megasynthase genes mbtB–F were induced upon infection and so were the majority of additional genes involved in mycobactin biosynthesis: mbtG/I/J/K/N (BCG2392c, BCG2400c, BCG2399, BCG1409c, BCG1408; Rv2378c, Rv2385, Rv2386c, Rv1347c, Rv1346) (Data set S1).

The type VII secretion system ESX-3 is essential for mycobactin-mediated iron acquisition and *in vivo* survival [43]. The ESX-3 secretion system is regulated by ZuR (BCG2373; Rv2359) [44] and consists of 11 genes [45] of which 7 were induced upon infection (Data set S1). The repression of *zuR*, resulted in the induction of ESX-3. A siderophore transport system of *M. tuberculosis* consisting of MmpL4 and MmpS4 (BCG0489c, BCG0490c; Rv0450c, Rv0451c) is required for infection of mice [46]. Both *mmpL4* and *mmpS4* and two other genes encoding an inner membrane transporter for mycobactin *irtA/B* [47] (BCG1410/1411; Rv1348/1349) were induced (Data set S1). Of the bacterioferritins BfrA/B (iron storage proteins induced by IdeR), only *bfrB* (BCG3904; Rv3841) showed a significant decrease. A possible explanation could be the reduced availability of iron in the host, and thus less iron storage capacity is required.

### *M. bovis* BCG small RNAs

Small RNAs have only recently been discovered in Mycobacteria [48,49]. Although their function is mostly unclear, they can be present in large quantities [50]. In our study, differential expression was observed for 19 small RNAs (FDR < 0.05). High transcript levels of the small RNAs MTS0997, MTS1338 and MTS2823 were reported in chronically *M. tuberculosis*-infected mouse lungs [50]. We observed a significant (FDR < 0.05) induction of MTS2823 as well, although the fold change is small (logFC = 1.49) compared to other reports. The expression of MTS1338 was repressed in our study, and showed a small fold change (logFC = −2.03). DosR (BCG3156c; Rv3133c) induces the latter in *M. tuberculosis* upon hypoxia and infection [51]. The low expression (below 100 CPM) and lack of induction of *dosR* in our datasets, may explain why MTS1338 remained unchanged. We verified the RNA-seq data by qRT-PCR (Additional file 5). Whether the contrary expression of MTS1338 in *M. bovis* BCG and in *M. tuberculosis* during infection is critical for virulence remains to be defined.

### Host immune response to *M. bovis* BCG is AIM2 dependent

A pathway enrichment analysis using InnateDB [52] revealed that the THP-1 cells show distinct signs of infection (Table 2) since we identified numerous enriched pathways involved in immune response such as IFN-α/β signaling, IFN-**γ** signaling and RIG-I/MDA5-mediated induction of IFN-α/β pathways. Interferons (IFNs) are synthesized by the host upon infection and trigger the activation of its immune system. IFNs can be divided in three classes: type I IFNs (IFN-α, IFN-β, IFN-ε and IFN-ω), type II IFNs (IFN-γ) and type III IFNs [53].

**Table 2:** **Induced THP-1 pathways upon** ***M. bovis*** **BCG infection**

**Pathway name**	**Number of genes annotated in pathway**	**Number of induced genes**	**P-value**
IFN-α/β signaling	36	23	<1.0 × 10^−5^
IFN-γ signaling	28	16	<1.0 × 10^−5^
RIG-I/MDA5-mediated induction of IFN-α/β pathways	12	8	<1.0 × 10^−5^
Cytosolic DNA-sensing pathway	23	8	0.00018
Cholesterol biosynthesis	14	6	0.0033
Hepatitis C	71	14	0.00073
*Staphylococcus aureus* infection	11	5	0.00078
Steroid biosynthesis	11	5	0.00078
Iron uptake and transport	7	4	0.00096
Toxoplasmosis	60	12	0.00152

The ten most significantly induced pathways are shown.

Shah and colleagues [54] showed that virulent mycobacteria, such as *M. tube*rculosis inhibit IFN-β production and signaling, resulting in the inhibition of the activation of AIM2 (interferon-inducible protein). AIM2 is part of the inflammasome that recognizes cytosolic bacterial and viral DNA, thereby contributing to the host's defense. In contrast to virulent mycobacteria, nonvirulent mycobacteria such as *M. smegmatis*, induce AIM2 [54]. *M. bovis* BCG seems to respond similarly to other nonvirulent mycobacteria, as the transcription of the gene encoding AIM2 is highly induced (Data set S1) as well as the IFN-α/β signaling pathway and the cytosolic DNA-sensing pathway (Table 2).

### Host genes involved in glycolysis and ketogenesis are induced upon mycobacterial infection

Phagocytosis of pathogenic mycobacteria triggers the accumulation of lipid bodies in the host cell described as foamy phenotype [55]. Secretion of mycobacterial ESAT-6 is required to mediate this process by stimulating the uptake of glucose into the host cell, which might lead to increased glycolytic activity and elevated levels of acetyl-CoA, which in turn leads to the generation of D-3-hydroxybutyrate via ketogenesis [55]. Although *M. bovis* BCG lost the ESX-1 locus, a major virulence determinant of pathogenic mycobacteria that encodes the effector proteins ESAT-6 and CFP-10 [13,56], we observe increased expression of several glycolytic enzymes including HK3, GPI, PFKP, FBPI, GAPDH, and PGAM1 (Additional file 2). Moreover, we found genes of the ketogenesis pathway induced: ACAT2, HMGCS1, BDH2 and HMGCR. The latter gene encodes HMG-CoA reductase, which catalyzes the conversion of hydroxy-β-methylglutaryl CoA and leads to the synthesis of cholesterol and other sterols. BDH2 encodes 3-hydroxybutyrate dehydrogenase, which catalyzes the reversible conversion of acetoacetate to D-3-hydroxybutyrate. Subsequent steps that lead to mycobacteria-induced formation of lipid bodies in the host cell involve the activation of the anti-lipolytic G protein-coupled receptor GPR109A, which triggers adenylyl cyclase. The resulting decrease in host cyclic AMP levels leads to a decrease in phosphorylation of stored host lipids by protein kinase A (PKA), rendering them less vulnerable to lipolysis by hormone sensitive lipase (HSL), thus promoting the formation of lipid bodies [55]. Consistent with the attenuated *M. bovis* BCG strain, we do not observe a change in the expression of GPR109A, adenylyl cyclase, PKA and HSL, indicating that this part of the pathway leading to the formation of lipid bodies is not active in the host or that this response is regulated post-transcriptionally and therefore remains invisible using a transcriptome approach.

Taken together, despite the absence of the ESX-1 locus in *M. bovis* BCG, the host response regarding the initial steps of lipid body formation is similar to that of *M. tuberculosis*. Several pathogen factors including mycolic acids that were demonstrated to induce the foamy phenotype in macrophages [57], may therefore be required to reprogram the host for lipid build-up.

### Cholesterol is synthesized and iron losses are compensated by the host upon infection

Four out of seven genes in the THP-1 iron uptake and transport pathway were induced. The enzyme encoded by the induced gene HMOX1 encodes heme oxygenase 1, assigned to iron uptake and transport, catalyzes the rate-limiting step of heme degradation and is required to confer host resistance to mycobacterial infection in mice [58]. Among the other induced genes were FTH1 and FTL, encoding the heavy and light polypeptide of ferritin. This suggests that the THP-1 cells compensate for the loss of iron caused by *M. bovis* BCG, by taking up extra iron and degrading heme.

The THP-1 cholesterol biosynthesis pathway was induced, as six genes of this pathway, including HMGCR encoding the rate-limiting enzyme for cholesterol biosynthesis, were upregulated. We conclude that infected macrophages synthesize cholesterol to compensate for loss of this molecule caused by mycobacterial catabolism.

## Discussion

This study describes a deep sequencing approach towards the elucidation of mycobacterial and host cell gene expression profiles during intracellular infection. Initially we employed standard deep sequencing settings for eukaryotes to resolve the transcriptional profile of intracellular mycobacteria. Although this set-up allowed, to some extent, analyzing gene expression in *M. bovis* BCG, mycobacterial transcript coverage was insufficient. Increasing the sequencing depth was hindered by high sequencing costs, thereby preventing the method to become a broad application. Thus, we decided to enrich for mycobacteria during sample preparation of infected THP-1 cells. Indeed, the strategy applied greatly increased the coverage of the intracellular *M. bovis* BCG transcriptome. Although mycobacterial gene expression can be analyzed in non-enriched samples (215 genes, FDR < 0.05), implementation of enrichment greatly expanded the number of reliably identified differentially expressed genes by 71% (367 genes, FDR < 0.05).

Moreover, the sequencing depth simulation (Figure 2B) revealed that enrichment allowed identification of differentially expressed genes that would have been missed otherwise. Repasy and colleagues showed that for an *in vivo* infection setting with mice and *M. tuberculosis* the MOI ranges from 1 to 5 [3]. Therefore, when studying an *in vivo* infection, the enrichment might not be sufficient to obtain a similar sequencing depth as obtained in our *in vitro* study, for which an MOI of 10 was used. To determine whether enrichment introduced any bias into the datasets, we analyzed non-enriched and enriched samples of two independent biological infection replicates. Although the non-enriched and enriched samples comprised different numbers of intracellular *M. bovis* BCG reads, the transcriptomes of respective samples of both infection experiments correlated well as indicated by high linearity and correlation coefficients (Additional file 1). Additionally, the correlation of intracellular *M. bovis* BCG expression between the biological replicates of both enriched datasets was comparable with the non-enriched datasets (0.93 and 0.94, respectively), verifying that the enrichment procedure was repeatable and robust, and did not introduce any bias to the intracellular mycobacterial transcriptome. For host expression we identified a lower correlation between the non-enriched and enriched samples (0.57 for IF1 and IF1ER, and 0.70 for IF2 and IF2ER) (Additional file 1). Hence, the datasets of non-enriched samples were favored for analysis of the host transcripts in order to preserve accuracy of the transcriptional landscape during infection.

Our method is dependent on differential susceptibility to lysis of host and microbial cells and not on a molecular sequence capture or depletion method as previously described [11]. This has the added advantage that small RNAs can be detected and analyzed for differential expression. Additionally, our method is independent of mRNA polyadenylation, as e.g. MICROBEnrich simultaneously captures and removes polyadenylated mRNAs along with rRNAs. We consider this a critical point because previous findings support the existence of polyadenylated tracts in mRNA of mycobacteria [59] and other bacteria [60,61].

It has been shown previously that mycolic acid liposomes are phagocytized by murine macrophages, changing the morphology of the macrophages to foam-like cells accumulating cholesterol [57]. Although the cholesterol degradation pathway is induced at 24 hours post-infection (Figure 3A and Additional file 3), the *mce4* gene cluster and *hsdD* and *choD* were not induced. Nonetheless, these genes were expressed in both conditions and respective proteins could be already present before infection, ready for a situation when cholesterol becomes available. For *hsdD* and *choD*, it is tempting to speculate that alternative genes with a similar function exist, since these genes have been found dispensable for cholesterol degradation in mycobacteria [21].

Gene regulation of mycobacterial cholesterol catabolism involves a complex interplay between KstR, KstR2, and IdeR. KstR and KstR2 are the prime regulators of the genes depicted in Figure 3A. A clear distinction between their targets becomes apparent with KstR regulating the degradation of rings A and B and KstR2 regulating the degradation of rings C and D. In addition, genes in these regulons (*fadA5*, *fadD3*, *fadE30*, *echA20*) contain IdeR-binding sites in their upstream regions [33] and in some cases a profound overlap between these binding sites was observed. This implies that these genes can only be expressed upon removal of both types of repressors: IdeR under normal iron availability and KstR and KstR2 during cholesterol shortage (Figure 5). The upregulation of IdeR-dependent iron uptake systems can be caused not only by low iron availability inside the macrophage but also as a response to the NO-induced damage caused to iron-containing proteins [5]. HsaC, KshA, and the cytochromes Cyp125 and Cyp142 are iron-containing enzymes (and hence are susceptible to NO-induced damage) and lack of functionality of these enzymes leads to the accumulation of stable toxic catabolic intermediates, such as catechol derivatives and cholest-4-en-3-one [62-65]. The IdeR control of this pathway ensures that it is only expressed when the corresponding repair/replacement systems for iron-containing proteins are in place, therefore minimizing the risk of toxic intermediate accumulation.

It has been shown that aspartate functions as a major nitrogen reservoir in the host [36]. In line with this finding, we observed induction of aspartate utilizing enzymes (Figure 3B). Interestingly, we did not detect induction of the gene encoding the aspartate transporter *ansP1*, but induction of its homolog, *ansP2*. Earlier reports demonstrated that an *ansP1*-KO mutant fails to import aspartate *in vitro*, but shows wild-type behavior in either resting or activated macrophages, even though aspartate is a major nitrogen source in the host. Moreover, the absence of *ansA* induction suggests AnsP2 functions as an aspartate importer during infection in addition to its role as asparagine importer [37].

Rodríguez and colleagues cultivated *M. tuberculosis* H37Rv on even long-chain fatty acids and analyzed the transcriptome by RNA sequencing, observing a dormancy-related phenotype [66]. Although there are similarities between their *in vitro* model and our results, the induction of cholesterol catabolism, the methylcitrate cycle and aspartate metabolism are not captured using such a method, highlighting the differences between using an *in vitro* model based on even long chain fatty acids as opposed to studying intracellular infection directly.

## Conclusions

Dual RNA-seq allowed elucidation of the complex interplay between *M. bovis* BCG and THP-1 macrophages. The comparison of non-enriched and enriched ribosomal RNA-depleted sequencing libraries of two biological replicates from identical infection cultures, showed high correlation of sequencing reads without technical bias. Taken together, microbe-enriched dual RNA-seq is a powerful technology that enables the assessment of the global transcriptome of "low-number" intracellular microbes and their host as demonstrated by the simultaneous induction of *M. bovis* BCG cholesterol degradation genes and host cholesterol synthesis genes.

## Methods

### Bacterial strains and growth conditions

*M. bovis* BCG SSI 1331 (American Type Culture Collection, #35733) was grown in Middlebook 7H9 medium (Becton Dickinson) supplemented with 0.05% Tween 80, 0.2% glycerol, 10% albumin-dextrose-catalase supplement (Becton Dickinson) (7H9-ADC) or on Middlebrook 7H11 agar (Becton Dickinson) containing 0.2% glycerol and 10% oleic acid-albumin-dextrose-catalase enrichment (Becton Dickinson). Mycobacterial cultures were grown to the mid-log phase in 1 L roller bottles (450 cm^2^) at 37°C and 2 rpm. For CFU enumeration, serial dilutions were performed in phosphate-buffered saline containing 0.05% Tween 80 and plated on Middlebrook 7H11 agar. Plates were incubated at 37°C for 3–4 weeks prior to counting.

### Infection of the human macrophage-like cell line THP-1

The THP-1 cell line (American Type Culture Collection #TIB-202) was maintained in Roswell Park Memorial Institute medium 1640 supplemented with 10% fetal calf serum, 2 mM glutamine, 1 mM sodium pyruvate and 0.05 mM 2-mercapto-ethanol in a humidified 5% carbon dioxide atmosphere at 37°C. Estimated 4 × 10^6^ cells/well in a 6-well plate were differentiated for 24 h using culture medium containing 40 ng/ml phorbol 12-myristate 13-acetate. Cells were then washed with fresh culture medium and incubated for 48 h. *M. bovis* BCG was pelleted (3,200 rpm, RT, 10 min), washed twice with phosphate buffered saline and resuspended in THP-1 culture medium. THP-1 cells were infected with *M. bovis* BCG at a multiplicity of infection of 10. The *M. bovis* BCG culture medium suspension was added to the differentiated cells, centrifuged for 3 min at 800 rpm and subsequently incubated for 4 h. The infection mix was removed, cells were washed twice with pre-warmed PBS and incubated with fresh culture medium for 20 h prior to RNA extraction.

### RNA isolation and mycobacterial RNA enrichment of infected cells

Total RNA from mycobacterial cultures was prepared as previously described [67]. Extraction of total RNA from THP-1 cells was prepared with TRIzol reagent using glycogen as a carrier according to the suppliers’ recommendation (Life Technologies). Total RNA from *M. bovis* BCG infected THP-1 cells was isolated by abrasive particles in a reciprocal shaker with TRIzol [67]. Enrichment of mycobacteria from infected THP-1 cells was carried out by differential lysis of host and mycobacterial cells by guanidine thiocyanate (GITC).

Infected cells were washed with PBS at RT. Cold 4 M GITC was added to the monolayer and the cells were transferred to a 1.5-ml screw cap tube. After centrifugation the pellet was resuspended in residual GITC and mixed with 1 ml TRIzol containing 20 μg/ml linear acrylamide, followed by incubation for 5 min at RT. Bacteria were disrupted by bead beating (FastPrep Instrument; two cycles of 30s at maximum speed with cooling on ice between cycles). The sample was centrifuged for 1 min at 4°C/13,000 rpm and the supernatant was transferred to a 2-ml screw cap tube containing 200 μl chloroform, mixed and incubated at RT for 5 min. After centrifugation at 4°C/13,000 rpm for 10 min, RNA was extracted from the aqueous phase using the Qiagen RNeasy mini kit including an on-column DNase digestion (Qiagen). Quality and quantity of total RNA were assessed using an Agilent 2100 Bioanalyzer (Agilent Technologies) and a NanoDrop 1000 spectrophotometer (Kisker).

### Calculation of spike-in concentration

Total RNA of THP-1 cells and *M. bovis* BCG cultures was determined on the basis of cell counts and RNA isolation yield. As the intracellular copy number for a pathogen varies from species to species we assumed the most minimal infection rate of one mycobacterium per host cell. Therefore a cellular multiplicity of infection of 1:1 was used for mixing host and pathogen RNA, resulting in a ratio of 1000:1 total RNA, derived from the proportion of RNA abundance per cell between host and pathogen.

### RNA sequencing

The RNA-seq libraries were prepared according to the TruSeq RNA Sample Preparation v2 Guide (Illumina) without fragmentation and without size selection. Using an Agilent Bioanalyzer high sensitivity DNA kit, we confirmed that the average final library size was already approximately 350 bp without fragmentation; therefore the library insert fragmentation time at 94°C was set to 0 minutes, as no additional fragmentation step was required. Up to 98% of bacterial RNA consists of ribosomal RNA, which can prevent adequate coverage of a bacterial transcriptome, using RNA sequencing [68,69]. Therefore, the Gram-Positive Bacteria Ribo-Zero (Epicentre) rRNA Magnetic Removal Kit was used to remove bacterial rRNA from mycobacterial total RNA. For THP-1 total RNA the Ribo-Zero Magnetic Kit Human/Mouse/Rat was used, while depletion of rRNA from infections without enrichment as well as the spike-in experiment was done by a two-step procedure with both kits. The mycobacterial enriched total RNA preparations of infected cells were depleted with the Ribo-Zero Magnetic Gold Kit Epidemiology. All cDNA libraries were checked for quality using the DNA-1000 kit (Agilent) on a 2100 Bioanalyzer and quantified with the Qubit 2.0 Fluorometer (Life Technologies). Libraries of each consecutive experiment were pooled as 4-plex and on-board loaded with a Hi-Seq 1500 instrument. The sequencing reaction was carried out as Rapid Run using a TruSeq Rapid PE Cluster Kit and a TruSeq Rapid SBS Kit and 2 × 51 cycles including 7 cycles indexing in order to obtain 50-bp paired-end reads.

### Data analysis pipeline

A total of eight datasets were created: Uninfected THP-1 cells (dataset: THP), exponentially growing *M. bovis* BCG (dataset: EGB), a spike-in sample consisting of THP-1 RNA and *M. bovis* BCG RNA in a 1000:1 ratio (dataset: SPI), *M. bovis* BCG-infected THP-1 cells with a multiplicity of infection of 10 *M. bovis* BCG bacilli per THP-1 host cell (dataset IF0), two additional independent biological replicates of *M. bovis* BCG-infected THP-1 cells (datasets: IF1 and IF2), and two datasets prepared with the enrichment method using the two biological replicates of *M. bovis* BCG-infected THP-1 cells (datasets: IF1ER and IF2ER).

All 50-bp paired-end reads from datasets IF1/2ER, IF0/1/2, EGB were aligned, using megablast, against the complete genome sequence of *M. bovis* BCG Pasteur, obtained from the NCBI bacterial genome database (ftp.ncbi.hih.gov/genomes/Bacteria) and to the human transcriptome obtained from the Ensemble database (www.ensembl.org). If both 50-bp reads of a given pair could not be aligned to the human transcriptome, or the *M. bovis* BCG genome, they were discarded from further analysis. The 50-bp paired-end reads aligning to the *M. bovis* BCG genome were subsequently aligned to protein-coding genes, ribosomal genes, and small RNA (sRNA) genes. The protein-coding gene sequences and the ribosomal gene sequences were obtained from the NCBI database. The sRNA-coding gene sequences were obtained from the Bacterial Small Regulatory RNA Database [70] and supplemented with sRNA sequences from *M. tuberculosis* [51].

### Gene counting procedure

A single count was assigned to a transcript if a complete pair of reads aligned to the *M. bovis* BCG genome. If only one read of a pair of reads aligned to a given gene, also a single count was assigned to this transcript, assuming that the other read could align to an intergenic region, or another gene, due to the existence of operons. If a pair of reads would align to two different genes, a count was assigned to both genes, resulting in a total of two counts per pair of reads. For THP-1 cells, all reads assigned to different splice variants of the same gene were counted (one count for each aligned read) and summed.

### Differential gene expression analysis

The R package edgeR [71] was used for differential gene expression analysis of *M. bovis* BCG and THP-1 genes. Gene and protein functions have been extensively studied in *M. tuberculosis* and therefore the *M. tuberculosis* (strain H37Rv) orthologs of each *M. bovis* BCG gene were included. Low expression tags receiving less than 100 counts per million in two or more of the three datasets: EGB, IF1/2ER (or EGB, IF1/2), were excluded from differential expression analysis as described in the edgeR manual. Tags receiving more than 100 counts per million in the EGB dataset and less than 100 counts per million in either one or both replicates of IF1/2ER or IF1/2 were still used to account for large decreases in expression upon infection.

EdgeR is a Bioconductor package designed to identify significant changes between two or more groups, given that at least one of the groups has replicated measurements [71], which is the case for our experimental setup (Figure 1). The edgeR algorithm models the read counts associated to a gene using a negative binomial probability distribution. The variance of this distribution takes into account both the stochasticity of the sequencing process and the variability associated to biological variation. The common Biological Coefficient of Variance (BCV) measures the average dispersion in gene expression values associated to biological variability. We used EdgeR to compute the BCV of the corresponding samples prior to computing differential expression. For the BCG genes, we obtain a BCV of 0.16 from the IF1/2ER samples whereas for the THP-1 genes we obtained a BCV of 0.20 from the IF1/2 samples. We assumed that the BCV in datasets EGB and THP to be smaller than those in datasets IF1/2ER and IF1/2, due to the less stable and controlled conditions arising from infection as compared to standard culturing procedures. To compute differential gene expression for *M. bovis* BCG and THP-1 cells, we took a conservative approach and assigned the same BCV to datasets EGB and THP as those obtained from IF1/2ER and IF1/2, respectively. Differential expression was then computed for each gene using a pairwise exact testing procedure. The algorithm of Benjamini and Hochberg was used to control false discovery rates [72]. Protein-coding genes and sRNA-coding genes with an FDR < 0.05 were assumed to be differentially expressed.

For THP-1 cells, gene counts from dataset THP were compared to those of datasets IF1/2. Differential gene expression analysis was performed similar to *M. bovis* BCG. The low expression tags cutoff of 100 counts per million was normalized for the number of genes in the THP-1 genome. Afterwards, we used InnateDB and pathway enrichment analysis to identify induced host pathways [52].

### Sequencing depth simulation

Subsets of 50-bp sequencing reads, where both pairs aligned to the complete genome of *M. bovis* BCG, were taken randomly from dataset EGB and IF1ER. These subsets were of the same size (3.35 M) as the total number of sequencing reads from dataset IF2ER aligning to the *M. bovis* BCG genome in pairs (Figure 2A). From these two subsets, and from the paired-end sequencing reads of IF2ER aligning to the *M. bovis* BCG genome, random sets of reads were chosen of different sizes. For each different size, five random sets were generated. Differential gene expression for *M. bovis* BCG was determined using the method described above (data analysis pipeline) for every set. The mean and standard deviation of the number of differentially expressed genes was calculated (Figure 2B).

### Compendium of expression data

A compendium containing 565 two-color microarrays for *M. tuberculosis* (strain H37Rv) was obtained from literature [73] and most of these (454) captured the effect of 75 drugs targeting metabolic pathways [74,75] whereas 111 captured stress-induced dormancy in the wild-type and in DosR activation genes in KO mutants [76,77].

### Quantitative real time PCR

To verify the expression of several genes and small RNA's, their expression was determined using qRT-PCR. For 13 genes, we designed primers (Additional file 4) and quantified their relative expression to the reference gene: *rpoB* (BCG0716; Rv0667). This gene encodes the RNA polymerase β-subunit and is thought to be a housekeeping gene, which was used as such in previous qRT-PCR studies [78-80]. Fold changes in gene expression (Additional file 5) were calculated using the ΔΔCt, as described previously [81].

### Availability of supporting data

Raw sequence read data supporting the results of this article are available in the EMBL-EBI European Nucleotide Archive under the Accession No. PRJEB6552, http://www.ebi.ac.uk/ena/data/view/PRJEB6552.

## Additional files

Additional file 1:
**Comparison between non-enriched and enriched datasets.** The normalized counts of each gene for both, *M. bovis* BCG (A and B) and THP-1 cells (C and D), of the non-enriched datasets were plotted versus the enriched datasets. The Pearson's correlation coefficients are denoted by an r in the lower right corner of each plot. The correlations for *M. bovis* BCG genes between non-enriched and enriched datasets, are higher than those of the THP-1 genes. Additional file 2:
**Differential expression of** ***M. bovis*** **BCG genes.** Excel file with all expressed *M. bovis* BCG protein-coding genes, *M. bovis* BCG small RNA genes, THP-1 genes, induced THP-1 pathways and repressed THP-1 pathways. Additional file 3:
**Cholesterol degradation pathway.** Genes in green are induced upon infection (FDR < 0.05), genes in black show no differential expression. Dashed arrows represent multiple reactions. The degradation of the ring C and D side chain is based on homologous genes from *Rhodococcus equi*. AD: 4-androstenedione, ADD: 1,4-androstenedione, 9OHADD: 9-hydroxy-1,4, androstene-3-17-dione, 3-HSA: 3-hydroxy-9,10-seconandrost-1,3,5(10)-triene-9,17-dione 3,4-DHSA: 3,4-dihydroxy-9,10-seconandrost-1,3,5(10)-triene-9,17-dione 4,9 DSHA: 4,5-9,10-diseco-3-hydroxy-5,9,17-trioxoandrosta-1(10),2-diene-4-oic acid, HDD: 2-hydroxy-hexa-2,4-dienoic acid, HIP: 9,17-dioxo-1,2,3,4,10,19-hexanorandrostan-5-oic acid, 5OH-HIP: 5-hydroxy-methylhexahydro-1-indanone propionate. *In *M. tuberculosis* H37Rv, *cyp142* has the same function as *cyp125*. In *M. bovis* BCG, *cyp142* contains a single nucleotide deletion resulting in a premature stop codon and an inactive gene product. This gene (*cyp142a*) is two-fold induced upon infection. Additional file 4:
**Primers used for qPCR.** Word document containing 14 primer pairs used for qPCR of selected *M. bovis* BCG genes. Additional file 5:
**Differential expression of selected** ***M. bovis*** **BCG genes using qPCR.** Excel file with Ct numbers for a variety of selected genes and sRNAs of *M. bovis* BCG. Fold changes in expression were calculated for these genes and sRNAs. Additional file 6:
**Central carbon metabolism.** Genes in green are induced, and genes in red are repressed upon infection (FDR < 0.05), genes in black show no differential expression. The genes in the methylcitrate cycle are induced upon infection. *In *M. tuberculosis* H37Rv, *icl2* is fragmented into *aceAa* and *aceAb* and non-functional.
