# Supplementary figures and images for: Comprehensive insights into transcriptional adaptation of intracellular mycobacteria by microbe-enriched dual RNA sequencing
Source: BMC Genomics. 2015 Feb 5;16(1):34. doi: 10.1186/s12864-014-1197-2 (PMC4334782; doi:10.1186/s12864-014-1197-2)

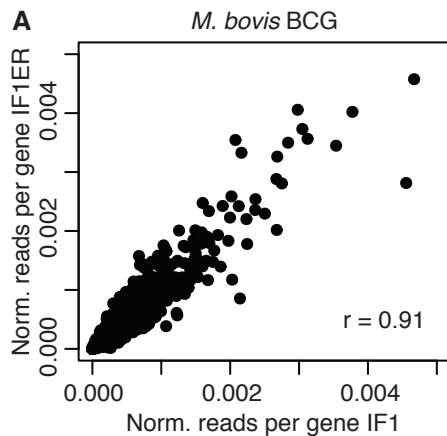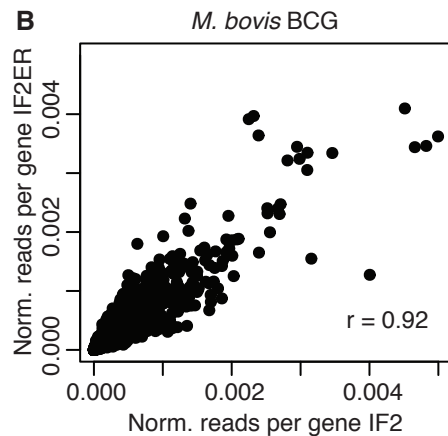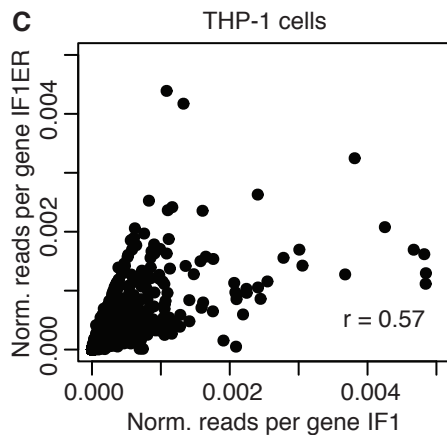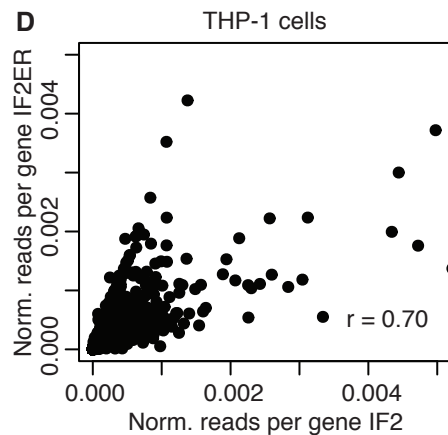

Supplement: Additional file 1: — Comparison between non-enriched and enriched datasets. The normalized counts of each gene for both, M. bovis BCG (A and B) and THP-1 cells (C and D), of the non-enriched datasets were plotted versus the enriched datasets. The Pearson's correlation coefficients are denoted by an r in the lower right corner of each plot. The correlations for M. bovis BCG genes between non-enriched and enriched datasets, are higher than those of the THP-1 genes. [file 12864_2014_1197_MOESM1_ESM.pdf]

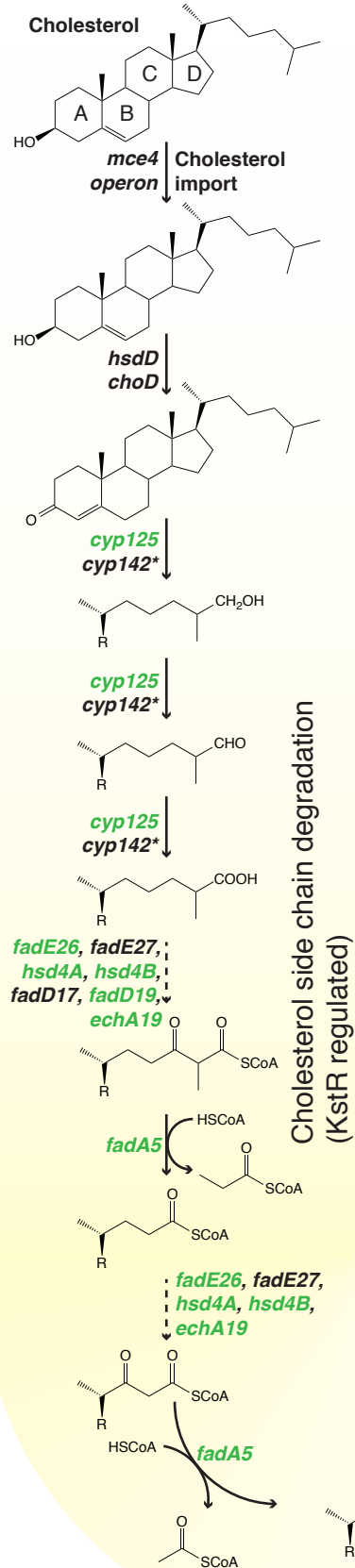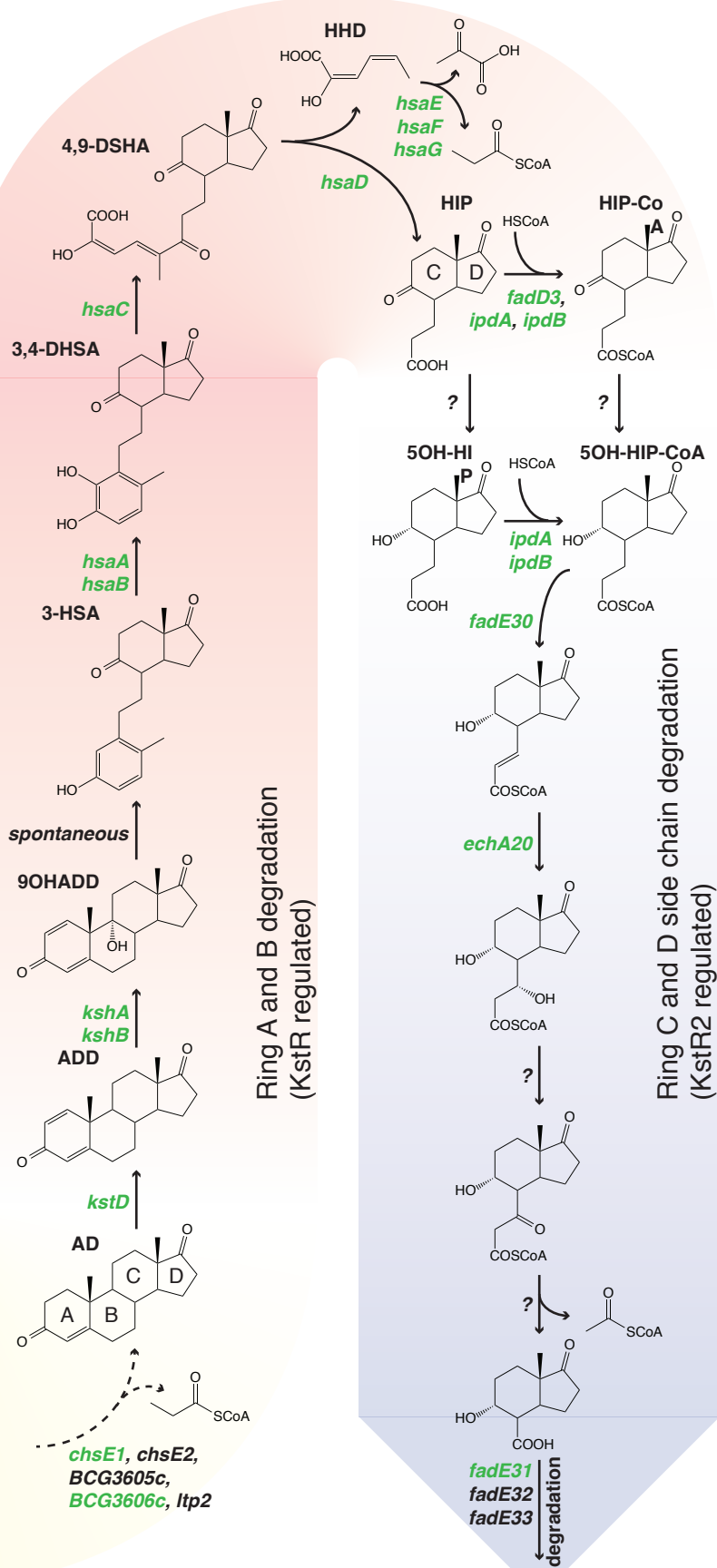

Supplement: Additional file 3: — Cholesterol degradation pathway. Genes in green are induced upon infection (FDR < 0.05), genes in black show no differential expression. Dashed arrows represent multiple reactions. The degradation of the ring C and D side chain is based on homologous genes from Rhodococcus equi. AD: 4-androstenedione, ADD: 1,4-androstenedione, 9OHADD: 9-hydroxy-1,4, androstene-3-17-dione, 3-HSA: 3-hydroxy-9,10-seconandrost-1,3,5(10)-triene-9,17-dione 3,4-DHSA: 3,4-dihydroxy-9,10-seconandrost-1,3,5(10)-triene-9,17-dione 4,9 DSHA: 4,5-9,10-diseco-3-hydroxy-5,9,17-trioxoandrosta-1(10),2-diene-4-oic acid, HDD: 2-hydroxy-hexa-2,4-dienoic acid, HIP: 9,17-dioxo-1,2,3,4,10,19-hexanorandrostan-5-oic acid, 5OH-HIP: 5-hydroxy-methylhexahydro-1-indanone propionate. *In M. tuberculosis H37Rv, cyp142 has the same function as cyp125. In M. bovis BCG, cyp142 contains a single nucleotide deletion resulting in a premature stop codon and an inactive gene product. This gene (cyp142a) is two-fold induced upon infection. [file 12864_2014_1197_MOESM3_ESM.pdf]

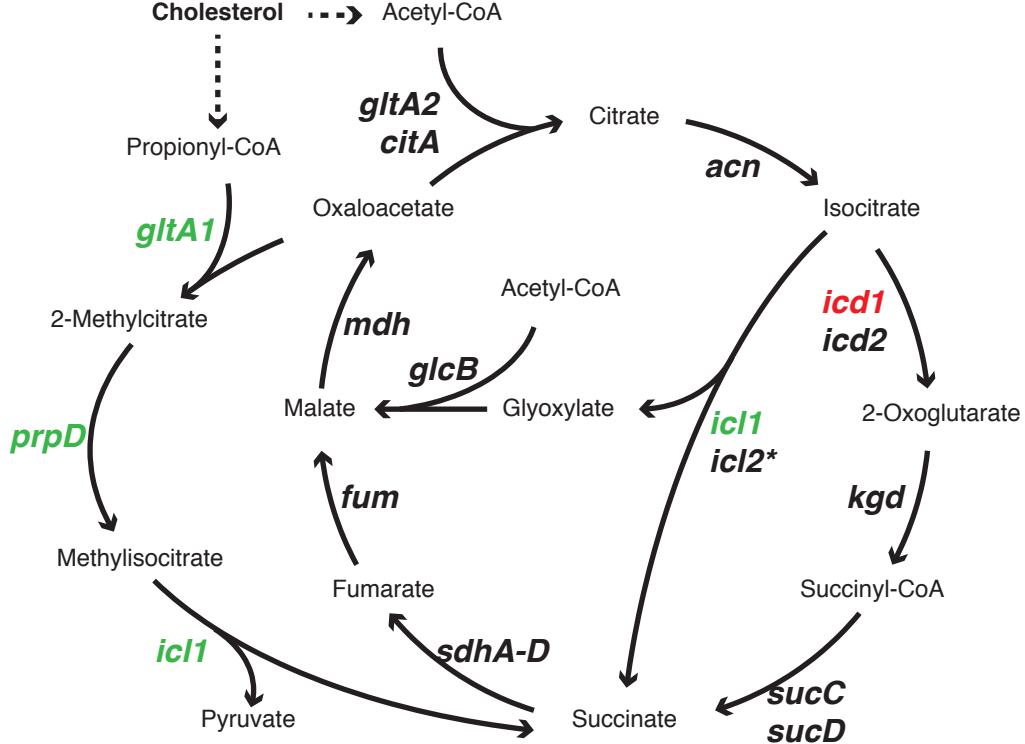

Supplement: Additional file 6: — Central carbon metabolism. Genes in green are induced, and genes in red are repressed upon infection (FDR < 0.05), genes in black show no differential expression. The genes in the methylcitrate cycle are induced upon infection. *In M. tuberculosis H37Rv, icl2 is fragmented into aceAa and aceAb and non-functional. [file 12864_2014_1197_MOESM6_ESM.pdf]
