# Supplementary material for: Comprehensive insights into transcriptional adaptation of intracellular mycobacteria by microbe-enriched dual RNA sequencing
Source: BMC Genomics. 2015 Feb 5;16(1):34. doi: 10.1186/s12864-014-1197-2 (PMC4334782; doi:10.1186/s12864-014-1197-2)
Supplement: Additional file 4: — Primers used for qPCR. Word document containing 14 primer pairs used for qPCR of selected M. bovis BCG genes. [file 12864_2014_1197_MOESM4_ESM.docx]

|  | Gene | Pathway | F/R | Sequence |
| --- | --- | --- | --- | --- |
| 1 | gltA1  BCG1192 | Methylcitrate cycle | F | AGCGATGGCTGGACATCTAC |
|  |  |  | R | CAGCCGGTGATCCTACTCAT |
| 2 | prpD  BCG1191 | Methylcitrate cycle | F | GGTCTGGTAACCGCCTATGA |
|  |  |  | R | ATCGCGTGGTAGATGGTCTC |
| 3 | glcB  BCG1872c | Glyoxylate shunt | F | GAGGTGTTCGAAGGCATCAT |
|  |  |  | R | AACCCCAGCACATCTTCAAC |
| 4 | kstR2  BCG3621c | Regulation cholesterol degradation | F | GGCAGCCTGTATCACCATTT |
|  |  |  | R | CGGTCCTCGATGTAGGAAAA |
| 5 | kstD  BCG3601 | Cholesterol degradation | F | TGTGGTGGTTTCGAGCATAA |
|  |  |  | R | AAGCGTCATCCATCAGATCC |
| 6 | hsaA  BCG3635c | Cholesterol degradation | F | GTGGTACGTGGTCGGCTTAC |
|  |  |  | R | TCGTAGTGGGATGCATTGTG |
| 7 | ipdA  BCG3615 | Cholesterol degradation | F | CTACTACGGGTTCGTCTCGC |
|  |  |  | R | CGTCGAAGTAGGGGTCGATG |
| 8 | fadD3  BCG3625 | Cholesterol degradation | F | CTACCTGTGCATCAACCCGT |
|  |  |  | R | CTCCGTGTCGTCCAGATAGC |
| 9 | nadA  BCG1632 | Quinolinate biosynthesis | F | CGTCGTTTCCTACGTCAACA |
|  |  |  | R | GTCCGGACAGAACAACACCT |
| 10 | mtbI  BCG2400c | Mycobactin biosynthesis | F | TCGTGATGACCTGGAATCAA |
|  |  |  | R | GATGCAGTGACAGCAGGAAA |
| 11 | MTS2823  smbb4074.1 | sRNA | F | GTTGCGGGTCTGCGTAATTG |
|  |  |  | R | GCTTCTAGCTGTAGCCCCAC |
| 12 | MTS1338  - | sRNA | F | GCTGTGTTTGGTGGCAGTATT |
|  |  |  | R | GTCTTGGGACGGTAGGTCAA |
| 13 | MTS1635 | sRNA | F | CGACCGACCAATAGGGTGAG |
|  |  |  | R | ATAATCACATCGGCCACGGG |
| 14 | rpoB  BCG0716 | RNA polymerase subunit beta | F | CGAGATCGTGTACCTGACCG |
|  |  |  | R | TAGTCCACCTCAGACGAGGG |

F/R: Forward or reverse primer
